# Venous and Arterial Endothelial Cells from Human Umbilical Cords: Potential Cell Sources for Cardiovascular Research

**DOI:** 10.3390/ijms22020978

**Published:** 2021-01-19

**Authors:** Skadi Lau, Manfred Gossen, Andreas Lendlein, Friedrich Jung

**Affiliations:** 1 Institute of Active Polymers and Berlin-Brandenburg Center for Regenerative Therapies, Helmholtz-Zentrum Geesthacht, 14513 Teltow, Germany; skadi.lau@hzg.de (S.L.); manfred.gossen@hzg.de (M.G.); 2 Institute of Chemistry, University of Potsdam, 14476 Potsdam, Germany

**Keywords:** human umbilical cord-derived endothelial cells, cardiovascular research, cardiovascular implants, artery endothelial cell compatibility testing

## Abstract

Although cardiovascular devices are mostly implanted in arteries or to replace arteries, in vitro studies on implant endothelialization are commonly performed with human umbilical cord-derived venous endothelial cells (HUVEC). In light of considerable differences, both morphologically and functionally, between arterial and venous endothelial cells, we here compare HUVEC and human umbilical cord-derived arterial endothelial cells (HUAEC) regarding their equivalence as an endothelial cell in vitro model for cardiovascular research. No differences were found in either for the tested parameters. The metabolic activity and lactate dehydrogenase, an indicator for the membrane integrity, slightly decreased over seven days of cultivation upon normalization to the cell number. The amount of secreted nitrite and nitrate, as well as prostacyclin per cell, also decreased slightly over time. Thromboxane B2 was secreted in constant amounts per cell at all time points. The Von Willebrand factor remained mainly intracellularly up to seven days of cultivation. In contrast, collagen and laminin were secreted into the extracellular space with increasing cell density. Based on these results one might argue that both cell types are equally suited for cardiovascular research. However, future studies should investigate further cell functionalities, and whether arterial endothelial cells from implantation-relevant areas, such as coronary arteries in the heart, are superior to umbilical cord-derived endothelial cells.

## 1. Introduction

Cardiovascular diseases, such as coronary artery disease affecting the heart, stroke affecting the brain, peripheral artery disease affecting the extremities, and aortic disease affecting all inner organs, represent the number one cause of death worldwide [1]. To restore blood flow in constricted vessels, cardiovascular devices such as stents or vascular grafts are often implanted. Hereby, mostly arteries are affected [2]. Since the coverage with endothelial cells (EC), which form a confluent monolayer on the inner side of all blood vessels, plays a critical role in the success of implants, much research has been conducted on the performance of this cell type on implant surfaces. Human umbilical vein endothelial cells (HUVEC) have represented the most common EC type used for in vitro endothelial cell research since 1973, due to the simple, quick, and inexpensive isolation process [3]. Results of HUVEC-based studies are often assumed to be transferrable to other EC types, such as arterial EC from different regions of the human body. However, a growing body of evidence shows that the endothelium is highly heterogeneous depending on the vascular bed, and that arterial EC differ from venous EC. As early as 1988, Wagner et al. showed that EC from bovine aortas differ from those of the venous circulation in terms of size, thickness, proliferation, and protein synthesis [4]. Arterial EC are typically thicker, and exhibit a rather long and ellipsoidal morphology compared to shorter and wider venous endothelial cells. This difference is probably due to different shear stress levels, which are higher in arteries than in veins (reviewed in [5,6]). Moreover, the molecular gene signature differs, as was shown by the identification of eight transcription factors that co-determine the arterial fingerprint in arterial EC compared to venous EC [7]. Today, two main identification markers, namely EphrinB2 for arterial EC, and EphB4 for venous EC, have been established [8]. Considering these differences it seems likely that arterial and venous EC also differ in their functions. It is known that the control of vascular tone is mainly attributed to arterioles. In contrast, post-capillary venules are the primary site of permeability and leukocyte trafficking during inflammation (reviewed in [5]). The formation of new blood vessels from existing ones, referred to as angiogenesis, occurs in both arteries and veins. Interestingly, this process is equally supported by vascular endothelial growth factor, but differentially inhibited by endogenous opioid growth factor [9].

Apart from these differences between venous and arterial EC, umbilical cord-derived EC represent a special case compared to EC located in the systemic circulation. In the latter, it is the function of arteries to transport oxygen- and nutrient-rich blood to organs, while veins deport oxygen- and nutrient-poor blood away from organs. In the umbilical cord, this situation is reversed. The umbilical cord-derived vein provides the fetus with oxygen and nutrients, while waste products are transported back to the mother through the arteries [10]. This raises the question of whether HUVEC could be considered as arterial EC and vice versa. A DNA microarray-based study comparing EC from various sources pointed against this hypothesis by showing that HUVEC cluster with EC from other veins, while HUAEC cluster with EC from other arteries in the systemic circulation [11]. Nevertheless, we asked ourselves whether HUVEC are actually a suitable cell type in the context of cardiovascular device testing, which are predominantly implanted in arteries or used to replace arteries. Instead, arterial cells from regions of the body that are targets of cardiovascular therapies, such as coronary arteries in the heart, might be superior to venous EC. However, EC from these sources are rarely available. In contrast, umbilical cords, each of which normally contains one vein and two arteries, are medical waste, and thus available in high numbers. A few recent studies chose human umbilical arterial endothelial cells (HUAEC), possibly anticipating that study results obtained with these cells are closer to arterial application areas than those obtained with HUVEC [12,13]. However, we are not aware of any study that has systematically analyzed HUVEC and HUAEC side-by-side for the characteristic properties of endothelial cells necessary for cardiovascular therapies. Endothelialization of implants requires the adhesion and proliferation of viable EC. Moreover, adherent EC need to be functional, with regard to the secretion of vasoactive substances such as nitric oxide, prostacyclin, and thromboxane, in order to control vasomotor tone, and to prevent platelet adhesion. In addition, the production of extracellular matrix proteins is important for enabling remodeling processes. Thus, it is the aim of the present study to compare HUVEC and HUAEC regarding cell viability, cell density, metabolic activity, membrane integrity, actin fiber patterns, secretion of vasoactive substances, and the production of von Willebrand factor, as well as of extracellular matrix proteins (collagens and laminin).

## 2. Results

**Figure 1:**
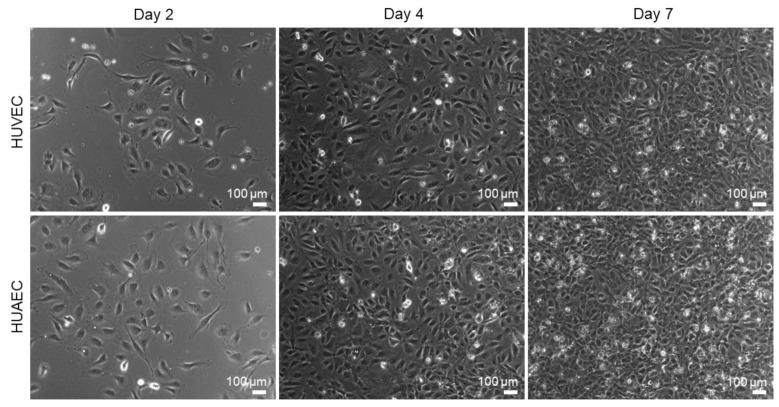
Morphology of endothelial cells. Human umbilical cord-derived endothelial cells from veins (HUVEC) or arteries (HUAEC) were seeded at a density of 15,000 cells/well at day 0, and cultivated for 2, 4, and 7 days. Phase contrast images were taken at 10-fold primary magnification. Cells developed the typical cobblestone-like morphology of mature endothelial cells with increasing proliferation time. Scale bar represents 100 µm.

**Figure 2:**
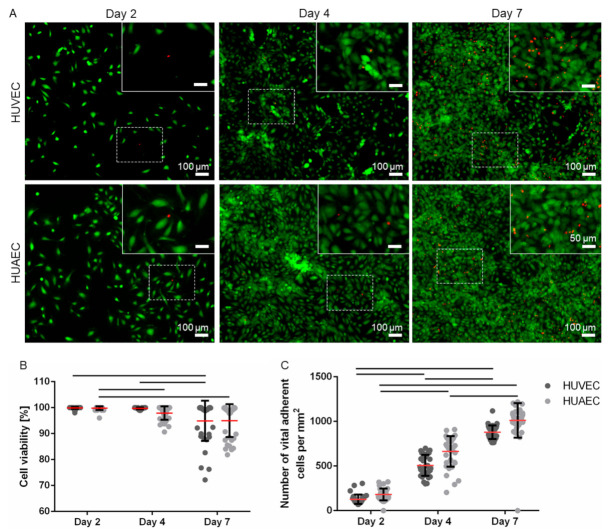
Endothelial cell viability and density. Human umbilical cord-derived endothelial cells from veins (HUVEC) or arteries (HUAEC) were seeded at a density of 15,000 cells/well at day 0, and cultivated for 2, 4, and 7 days. Cell viability staining detected living (green) and dead (red) cells (**A**). Quantification of vital cells showed that only a few cells died over time (**B**). The cell density, expressed as the number of vital adherent cells per mm^2^, increased over time (**C**). Fluorescent images were taken at 10-fold primary magnification. Scale bar represents 100 µm, and 50 µm in the magnified image sections in the upper right corner of each image. Squares with dashed lines indicate the image region that was magnified. Straight black lines indicate *p* ≤ 0.0099.

HUVEC are the most widely used cell type for in vitro endothelial cell research. The present study was conducted to explore the use of HUAEC as a potential alternative to HUVEC. For this, both cell types were cultivated for seven days and analyzed regarding several endothelial cell-specific properties. Phase contrast images revealed that the cell morphology of both cell types was similar (Figure 1). While few of the EC showed the characteristic morphology at day 2 of cultivation, rather displaying a roundish to elongated shape, they phenotypically shifted towards a typical cobblestone-like pattern with increasing cell density at day 4 and 7. Analysis of cell viability (FDA/PI-assay) revealed a moderate increase over time in the fraction of dead cells, as indicated by the presence of mainly viable/green cells and few dead/red cells (Figure 2A). At day 2, 99.9% ± 0.4% (HUVEC), and 99.8% ± 0.7% (HUAEC) of the cells were viable. At day 4, cell viability decreased only marginally to 99.7% ± 0.4% (HUVEC), and 97.9% ± 2.6% (HUAEC). A more substantial decrease of cell viability was detected at day 7, however, in a comparable manner for both cell types. Among HUVEC, 94.9% ± 7.8% of cells were viable, while among HUAEC, 95.0% ± 6.4% were viable (Figure 2B). No statistical differences were found between HUVEC and HUAEC, with regard to cell viability and also cell density, which increased with both HUVEC and HUAEC in a similar fashion over time (Figure 2C).

**Figure 3:**
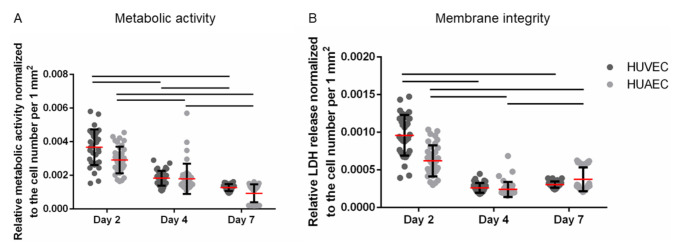
Metabolic activity (**A**) and membrane integrity (**B**) of endothelial cells. Human umbilical cord-derived endothelial cells from veins (HUVEC) or arteries (HUAEC) were seeded at a density of 15,000 cells/well at day 0 and cultivated for 2, 4, and 7 days. The metabolic activity and membrane integrity were normalized to the number of vital adherent cells per mm^2^ leading to decreasing values over time. Straight black lines indicate *p* ≤ 0.0058.

The MTS assay is based on the enzymatic reduction of a yellow tetrazolium salt into red formazan by NAD(P)H-dependent dehydrogenases, a reaction that is restricted to metabolically active, viable cells. Thus, this method assesses both metabolic activity and cell viability. Moreover, it is often used to determine proliferation, since the generation of formazan increases with increasing cell numbers. Here, the metabolic activity was normalized to the number of vital adherent HUVEC and HUAEC to compensate for cell count-dependent differences. While absolute numbers per well increased over time, upon normalization a slight reduction in signal per cell was observed. No statistically significant differences were detected between the two cell types at any time point (Figure 3A). The same was true for the membrane integrity, as assessed by the release of lactate dehydrogenase (LDH). While absolute numbers per well increased up to day 7, though not as much as with the mitochondrial activity, data normalization resulted in a slight decrease of the LDH release over time. However, no statistically significant differences were found between HUVEC and HUAEC (Figure 3B).

**Figure 4:**
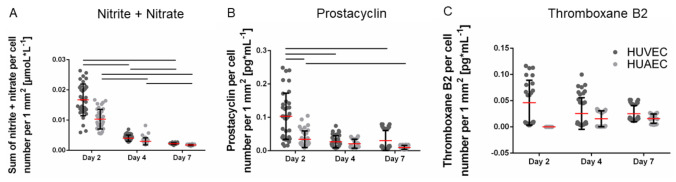
Secretion of vasoactive substances by endothelial cells. Human umbilical cord-derived endothelial cells from veins (HUVEC) or arteries (HUAEC) were seeded at a density of 15,000 cells/well at day 0 and cultivated for 2, 4, and 7 days. The amounts of the sum of nitrite and nitrate (**A**), prostacyclin (**B**), and thromboxane B2 (**C**) were quantified by ELISA and normalized to the number of vital adherent cells per mm^2^. While less nitrite/nitrate and prostacyclin were released over time, the amount of thromboxane B2 remained constant. Straight black lines indicate *p* ≤ 0.0063.

The production of vasoactive substances is a characteristic property of functional endothelial cells. The total amount of secreted nitrite and nitrate per well, two stable metabolites of nitric oxide, remained constant over the seven days of cultivation. Upon normalization to the cell number, which increased over time, the amount of nitrite and nitrate per cell decreased. The absolute amounts of prostacyclin increased up to day 7, though only marginally. Consequently, the amount of secreted prostacyclin per cell slightly decreased over time. However, no significant differences were observed between HUVEC and HUAEC except for the prostacyclin production at day 2, which was slightly reduced in the case of HUAEC compared to HUVEC (Figure 4A,B). In contrast, the total amount of thromboxane B2 increased over time, resulting in constant values upon normalization to the cell number (Figure 4C). At day 2, HUAEC did not produce any thromboxane B2. At day 4 and day 7, no differences were visible between HUVEC and HUAEC.

**Figure 5:**
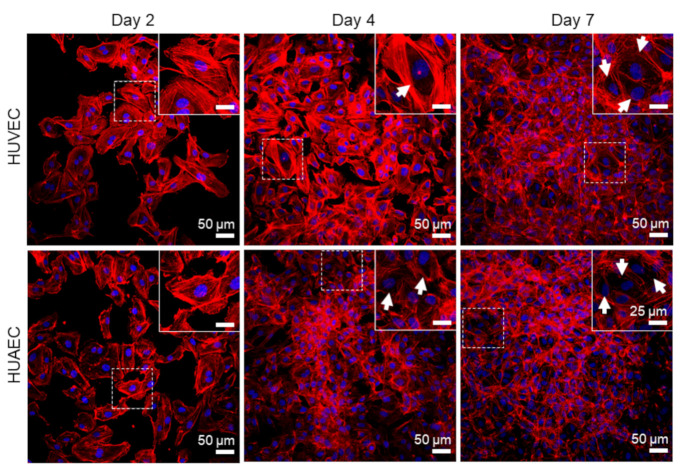
Expression of actin fibers by endothelial cells. Human umbilical cord-derived endothelial cells from veins (HUVEC) or arteries (HUAEC) were seeded at a density of 15,000 cells/well at day 0 and cultivated for 2, 4, and 7 days. While most of the cells exhibited actin fibers throughout the cell body at day 2, an increasing number of cells with pronounced cortical actin fibers were present at day 4 and 7 (indicated by white arrows). Fluorescent images were taken at 20-fold primary magnification. Scale bar represents 50 µm, and 25 µm the in magnified image sections in the upper right corner of each image. Squares with dashed lines indicate the image region that was magnified.

**Figure 6:**
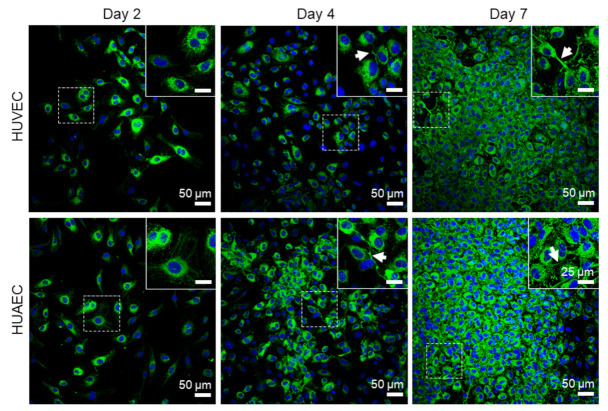
Expression of von Willebrand factor (vWF) by endothelial cells. Human umbilical cord-derived endothelial cells from veins (HUVEC) or arteries (HUAEC) were seeded at a density of 15,000 cells/well at day 0 and cultivated for 2, 4, and 7 days. Cells from both sources expressed vWF at all time points. At day 4 and 7, some extracellular vWF fibers were observed (white arrows). Fluorescent images were taken at 20-fold primary magnification. Scale bar represents 50 µm, and 25 µm in the magnified image sections in the upper right corner of each image. Squares with dashed lines indicate the image region that was magnified.

**Figure 7:**
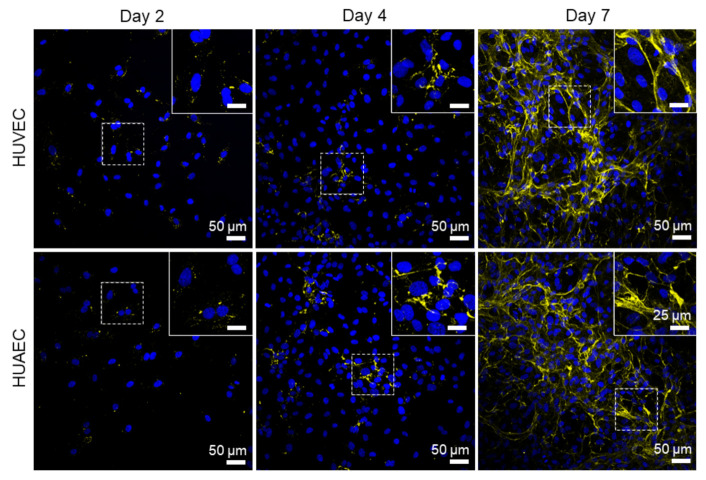
Expression of collagen I-V by endothelial cells. Human umbilical cord-derived endothelial cells from veins (HUVEC) or arteries (HUAEC) were seeded at a density of 15,000 cells/well at day 0 and cultivated for 2, 4, and 7 days. While only traces of collagen were detected at day 2, the expression increased with increasing cultivation time, leading to large networks of collagen fibers at day 7 with cells from both sources. Fluorescent images were taken at 20-fold primary magnification. Scale bar represents 50 µm, and 25 µm in the magnified image sections in the upper right corner of each image. Squares with dashed lines indicate the image region that was magnified.

**Figure 8:**
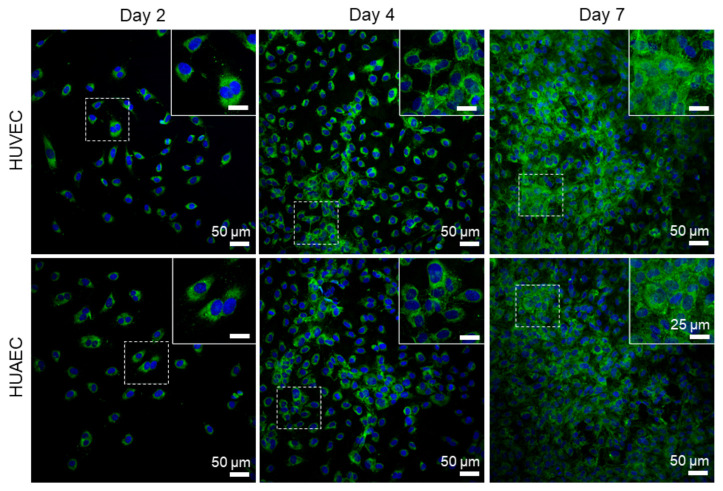
Expression of laminin by endothelial cells. Human umbilical cord-derived endothelial cells from veins (HUVEC) or arteries (HUAEC) were seeded at a density of 15,000 cells/well at day 0, and cultivated for 2, 4, and 7 days. While laminin was only intracellularly present at day 2, it was secreted into the extracellular space with increasing cultivation time, leading to a wide fleece-like network at day 7. Fluorescent images were taken at 20-fold primary magnification. Scale bar represents 50 µm, and 25 µm in the magnified image sections in the upper right corner of each image. Squares with dashed lines indicate the image region that was magnified.

Finally, the presence of actin fibers, vWF, and extracellular matrix molecules, collagen and laminin, was evaluated by antibody-staining. In general, no differences were detected between HUVEC and HUAEC. Actin fibers were observed at all time points, however, the localization in the cells slightly differed depending on the time point. For example, at day 2, actin fibers in most cells pervaded the whole cell body. This was also observed at day 4 and 7, however, at these time points, an increasing number of cells also showed cortical actin filaments and an actin fiber-free cytoplasmic area (Figure 5, white arrows). vWF was mainly present intracellularly at all time points, but some vWF fibers were also detected in the extracellular space at day 4 and 7 (Figure 6, white arrows). The production of collagen and laminin increased over the 7 days of cultivation. Traces of collagen were visible at day 2 in the form of small little dots, which were most likely inside the cells. At day 4, some short fibers were observed, and at day 7 large networks of collagen fibers were present throughout the cell layer, indicating the secretion of collagen into the environment (Figure 7). Similarly, laminin was detected within the cells at day 2 and day 4. With increasing cell density, thin laminin strands connecting two or more cells were observed. At day 7, a dense laminin-based fleece seemed to cover the substrate underneath the cells (Figure 8).

## 3. Discussion

Cardiovascular implants are most frequently implanted to restore arterial functions. However, most studies have been performed with venous EC. To evaluate whether differences between umbilical cord-derived venous and arterial EC exist regarding selected properties necessary for cardiovascular implants, the present study compared cells from both sources side-by-side. The major finding is that no differences in either of the tested parameters were found between EC from both sources.

Proliferation of cells from both sources was comparable over the seven days of cultivation. This is in agreement with a study by Rosa et al., who compared iPSC-derived EC, HUVEC, and HUAEC on three different substrates [14]. However, the general opinion is that EC proliferation in veins is higher than in arteries [15]. The use of specific proliferation markers, such as Ki67, and assays based on DNA replication, such as the BrdU assay, might be useful in future studies to investigate this issue in detail. On the other hand, there is increasing evidence for the heterogeneity of the endothelium depending on the vascular bed, the developmental stage, and the microenvironment. In blood, different types of endothelial progenitor cells exist, which can be differentiated by their high proliferative capacity [16]. These progenitors were also found in the wall of mature arteries and veins, explaining why HUVEC and HUAEC can be cultivated to a comparable number of population doublings like progenitor cells [17]. Since the proliferation capacity of progenitor cells is greater than that of mature EC, HUVEC and HUAEC might have been overgrown by these progenitor cells resulting in similar proliferation behaviors. To investigate this issue, progenitor cells could be identified by their clonogenic potential in single cell colony forming assays. Moreover, cells could be analyzed regarding endothelial progenitor markers, such as CD34 or CD133, which should be absent in mature EC [18].

The metabolic activity differs between arterial, venous, microvascular, and lymphatic EC in terms of glucose and oxygen consumption, the amount of intracellular ATP levels and other parameters (reviewed in [19]). With regard to umbilical cord-derived EC, the metabolism of HUVEC was mostly studied, while HUAEC were largely neglected. For example, Lorenz et al. found that serum starvation and treatment with vascular endothelial growth factor induced higher intracellular ATP levels in female HUVEC than in male cells, indicating an energetic advantage for female cells over male cells [20]. Another study revealed that the metabolic activity increased with increasing cultivation time as assessed by enzymes involved in glycolysis and the citric acid cycle [21]. One study reported both HUVEC and HUAEC to have a similar glycolytic flux (reviewed in [22,23]). This is in agreement with the present study, which also detected a similar metabolic activity by MTS assay, a measure of the glycolytic activity of cells.

A confluent monolayer of intact EC in the blood vessel lumen ensures a selective permeability for molecules and cells. An impaired membrane integrity, e.g., measured by an increased release of lactate dehydrogenase, would reduce barrier function. For example, necrotic and late apoptotic cells exhibit a compromised membrane integrity compared to healthy cells [24]. Likewise, inflammatory molecules, such as tumor necrosis factor α or hyperglycemic conditions, as present in diabetes, can also change the integrity [25,26]. Thus, it is not surprising that, in the present study, low passage HUVEC and HUAEC cultivated without any disturbing factors show a comparably intact membrane integrity. Future studies using fluorescent dextran diffusion or staining of inter-endothelial junctions might reveal additional insights regarding the endothelial barrier function of venous and arterial EC.

The secretion of vasoactive substances by EC has been analyzed in multiple studies. Nitric oxide (NO) and prostacyclin are important vasodilators, leading to the relaxation of smooth muscle cells and a reduction of blood pressure. In contrast, thromboxane A2 (here the more stable variant thromboxane B2 was measured) induces vasoconstriction and an increase of blood pressure. Several studies have investigated these parameters in vitro and in vivo, with different results. Fukaya et al. found that EC of canine arteries released more NO than EC of veins in response to acetylcholine and flow [27]. In contrast, cultured EC from porcine femoral artery and vein showed a comparable production of NO after stimulation with lipopolysaccharide. However, prostacyclin secretion was lower in venous cells compared to arterial cells [28]. An early study found that bovine arterial and venous EC produce similar amounts of prostacyclin and NO [29]. With regard to thromboxane, and in the absence of stimulators, it was shown that HUVEC, which are macrovascular cells, produce less thromboxane compared to microvascular EC from the placenta or foreskin. On the other hand, no differences in the prostacyclin production were found [30]. In the present study, the secretion of all three tested substances was similar for HUVEC and HUAEC. Considering the different results of the aforementioned studies, none of which compared human macrovascular EC, it can be speculated that the production of vasoactive molecules is highly dependent on the species, the classification as macro- or microvascular EC, and most likely also on culture conditions. Moreover, one has to keep in mind that a comparison of different studies is complicated by the use of different stimulators, which differently regulate the production of vasoactive substances.

Actin fibers, as part of the cytoskeleton, were observed in EC from both sources at all time points. While these fibers pervaded the whole cell body of most cells at day 2, they shifted towards the outer cell boundary at day 4 and day 7. This indicated the formation of a functionally confluent endothelial monolayer, important for mediating anti-thrombogenic actions [31]. The von Willebrand factor (vWF) is a key player in hemostasis, stored in Weibel–Palade bodies in EC, and released both constitutively at low concentrations, and at events of inflammation in high amounts (reviewed in [32,33]). For example, EC activation by high concentrations of acrolein, a metabolite of the anti-cancer drug cyclophosphamide, induces a complete loss of vWF in HUVEC [34]. While cultivation of EC under shear stress stimulates the elongation of secreted vWF molecules, few vWF fibers are released under static conditions [35]. In the present study, vWF largely remained intracellularly in EC from both sources, with few fibers in the extracellular space at all time points, indicating that no cell activation took place.

Collagen type IV, and laminin 411 and 511 (isotypes 8 and 10) are central components of the endothelial basal lamina, a specialized part of the extracellular matrix (reviewed in [36]). Surprisingly little is known about the spatial and temporal synthesis of these proteins in EC cultured in vitro. Kusuma et al. compared the production and release of collagen type I and IV, as well as laminin (isotype not specified), by endothelial progenitor cells and mature EC, such as HUVEC and HUAEC cultivated at three different oxygen concentrations (1% (*v*/*v*), 5% (*v*/*v*), and 21% (*v*/*v*) O_2_), and at three different days of cultivation (1, 4, and 7 days). At 21% (*v*/*v*) O_2_, only endothelial progenitor cells were able to produce collagen type IV at all time points. All three cell types produced laminin, which remained intracellularly in mature EC, but formed a network of fibers in the case of progenitor cells. Collagen type I was not detected. At 1% and 5% (*v*/*v*) O_2_, all three EC types deposited a fibrous mesh of collagen type IV and laminin in the extracellular space. Collagen type I was yet again not detected [37]. The present study, using a pan-specific collagen antibody, found that the assembly of a fibrous collagen network started at day 4, and was fully developed at day 7 in cultures of both HUVEC and HUAEC cultivated at 21% (*v*/*v*) O_2_. The same was true for laminin (isotype also not specified), which was increasingly secreted into the extracellular space with increasing cultivation time. In a previous study of our group, comparing arterial EC from different donors, collagen was also secreted at 21% (*v*/*v*) O_2_ either at day 4 or day 7, depending on the individual donor. However, laminin, which was detected with the same antibody as used in the present study, remained intracellularly in cells from all donors [38]. Thus, the synthesis and especially the secretion of ECM molecules may not be exclusively oxygen-dependent, but also donor-dependent.

## 4. Materials and Methods

### 4.1. Study Design

Human umbilical vein endothelial cells (HUVEC) and human umbilical artery endothelial cells (HUAEC) were cultivated for 2 days, 4 days, and 7 days prior to the analysis of cell viability, cell density, metabolic activity, membrane integrity, secretion of vasoactive substances (nitric oxide, prostacyclin, and thromboxane B2), and expression of filamentous actin fibers and von Willebrand factor, as well as extracellular matrix proteins, such as collagen and laminin. In total, three independent experiments were performed with 9 to 36 technical replicates, depending on the assay.

### 4.2. Cell Culture

Pooled HUVEC (from 3 to 6 individual donors, Lonza, Cologne, Germany) and HUAEC (from 250 individual donors, PeloBiotech GmbH, Planegg, Germany) were cultivated in EGM-2 Bullet Kit containing endothelial cell culture medium supplemented with 2% (*v*/*v*) fetal bovine serum, vascular endothelial growth factor, basic fibroblast growth factor, human epithelial growth factor, insulin-like growth factor-1, hydrocortisone, heparin, ascorbic acid, gentamycin, and amphotericin B (Lonza) in a standard humidified incubator at 37 °C with 5% (*v*/*v*) CO_2_. Cells of passage 5 were seeded in 4-well PCA chamber slides (Sarstedt, Nürnbrecht, Germany) at a cell density of 15,000 cells/well with 1 mL medium per well, and cultivated for up to 7 days. Medium was exchanged at day 2 and day 4 of cultivation.

### 4.3. Cell Viability

Cell viability was assessed by fluorescein diacetate (FDA, 12.5 µg·mL^−1^, Invitrogen, Darmstadt, Germany) to stain viable cells green, and propidium iodide (PI, 1 µg·mL^−1^, Sigma-Aldrich, Taufkirchen, Germany) to stain dead cells red. For this, both FDA and PI were simultaneously added to the cell culture medium and images of stained cells were immediately taken in a 10-fold primary magnification (Axio Vert.A1, Zeiss, Jena, Germany). Numbers of viable and dead cells were counted using Fiji is just imageJ (version 1.48c) [39] and expressed in percent of the total cell number. Moreover, the cell density of viable adherent cells was normalized to the growth area, and expressed as the number of vital adherent cells per mm^2^ (*n* = 36; three independent experiments with 12 images per experiment, taken from four different wells).

### 4.4. Metabolic Activity

The metabolic activity of cells was assessed using a MTS cell Titer 96 Aqueous Non-radioactive Cell Proliferation Assay (MTS assay, Promega, Mannheim, Germany) according to the manufacturer’s instructions. Briefly, 100 µL of MTS-mixture was added to 500 µL cell culture supernatant in a 24-well plate (Th. Geyer, Berlin, Germany). After 3 h of incubation at 37 °C, the absorbance was measured at 492 nm using a photometer (Tecan infinite M200 pro, Crailsheim, Germany). The metabolic activity was normalized to the number of vital adherent cells per mm^2^ (*n* = 9; three independent experiments measured in triplicates).

### 4.5. Cell Membrane Integrity

The cell membrane integrity was measured using a LDH Cytotoxicity Assay Kit II (Roche, Grenzach, Germany) according to the manufacturer’s instructions. Briefly, 10 µL of cell culture supernatant was added to 100 µL of LDH reaction mix, and incubated for 30 min at room temperature in the dark. The absorbance was measured at 450 nm (reference wavelength: 650 nm) using a photometer (Tecan infinite M200 pro). The cell membrane integrity was normalized to the number of vital adherent cells per mm^2^ (*n* = 36; three independent experiments with three different wells per experiment, measured in four technical replicates).

### 4.6. Ultrafiltration and Lyophilization of Cell Culture Supernatants

To quantify the amount of vasoactive substances produced by HUVEC and HUAEC at day 2, day 4, and day 7 of cultivation, cell culture supernatants were collected at each time point. Since fetal bovine serum (FBS) can interfere with enzyme linked immunosorbent assays, it was removed from supernatants by ultrafiltration. For this, ultracentrifugation tubes (Amicon, Merck KGaA, Darmstadt, Germany) with 10 kDa filter membranes were wetted with ultra-pure water (Cayman Chemicals, Hamburg, Germany), and subsequently filled with cell culture supernatant. Tubes were centrifuged for 20 min at 4000× *g* and room temperature. Finally, the FBS-free supernatants were stored at −20 °C. Since the concentration of vasoactive substances in cell culture medium is relatively low, and often close to the detection limit, samples were lyophilized to increase the concentration of these substances, and allow their measurement in a reliable range of the standard curve. For this, frozen samples were lyophilized using an ALPHA 1-4 LD plus (Christ, Osterode, Germany) for four days. Finally, lyophilized samples were stored at −20 °C until quantification. These samples were rehydrated to one third of the original volume in ultra-pure water to prepare for analysis. This concentration factor (3) was taken into account during the calculation of the amount of secreted substances.

### 4.7. Quantification of Secreted Vasoactive Substances

#### 4.7.1. Nitric Oxide

Nitric oxide production by cells was indirectly measured by quantification of the more stable metabolites of nitric oxide, nitrate, and nitrite, using a commercial kit (Total nitric oxide and nitrate/nitrite Kit, R&D Systems, Wiesbaden, Germany) according to the manufacturer’s instructions. In the first step, the amount of endogenous nitrite was measured. In a second step, the amount of total nitrite, generated after enzymatic conversion of nitrate into nitrite by nitrate reductase, was colorimetrically detected as an azo dye product of the Griess reaction. To calculate the amount of nitrate, the endogenous nitrite was subtracted from the total nitrite. Technically, 50 µL cell culture supernatant was used per well, and measurements were performed in triplicates. Absorbance was recorded at 540 nm (reference wavelength: 690 nm) using a photometer (Tecan infinite M200 pro). All concentrations were calculated based on a standard curve and normalized to the number of vital adherent cells per mm^2^ (*n* = 9; three independent experiments, measured in triplicates).

#### 4.7.2. Prostacyclin and Thromboxane B2

Prostacyclin and thromboxane B2 secretion was quantified using two different competitive enzyme immunoassays (6-keto Prostaglandin F_1α_ ELISA Kit, and Thromboxane B2 ELISA Kit, Cayman Chemical, Hamburg, Germany) according to the manufacturer’s instructions. Briefly, 50 µL cell culture supernatant was used per well and measurements were performed in triplicates. Absorbance was recorded at 410 nm using a photometer (Tecan infinite M200 pro). Prostacyclin and thromboxane B2 concentrations were calculated based on a standard curve, and normalized to the number of vital adherent cells per mm^2^ (*n* = 9; three independent experiments measured in triplicates).

### 4.8. Fluorescence Staining of Actin Fibers, Von Willebrand Factor, Collagen and Laminin

To visualize filamentous actin fibers, von Willebrand factor, collagen, and laminin, an immunocytological staining was performed. For this, cells were fixed with 4% (*w*/*v*) paraformaldehyde (Sigma-Aldrich, Taufkirchen, Germany) for 30 min, permeabilized using 0.5% (*v*/*v*) Triton-X-100 for 10 min (Sigma-Aldrich, Taufkirchen, Germany), and unspecific binding sites were blocked with 5% (*w*/*v*) bovine serum albumin (Roth, Karlsruhe, Germany) for 20 min at room temperature. As primary antibodies, rabbit anti-human von Willebrand factor (Sigma-Aldrich, F3520, 1:200), rabbit anti-human collagen type I-V (Abcam, Cambridge, United Kingdom, ab36064, 1:250), and mouse anti-human laminin (Novus Biologicals, Wiesbaden Nordenstadt, Germany, NB600-883, 1:250) were incubated for 1 h at room temperature. After three washing steps with phosphate buffered saline (Thermo Fisher Scientific, Berlin, Germany) cells were incubated with the secondary antibodies, donkey anti-rabbit IgG conjugated to Alexa Fluor 488 (Thermo Fisher Scientific, A21206, 1:500, for vWF), donkey anti-rabbit IgG conjugated to DyLight 550 (Thermo Fisher Scientific, SA5-10039, 1:500, for collagen I-V), and donkey anti-mouse IgG conjugated to Alexa Fluor 488 (Thermo Fisher Scientific, A31570, 1:400, for laminin) for 1 h at room temperature in the dark. Finally, cells were washed and covered with ROTI^®^Mount FluorCare as mounting medium, containing 4′,6-diamidino-2-phenylindole (DAPI, Roth, Karlsruhe, Germany) to counterstain cell nuclei. For qualitatively analysis, images were taken in 20-fold primary magnification using a confocal laser scanning microscope (DMi8, Leica, Wetzlar, Germany) (*n* = 18; three independent experiments, where 2 images were taken per well from a total of three wells per experiment).

### 4.9. Statistics

Statistical analyses were performed using Graphpad Prism 6 (Graphpad Software, San Diego, CA, USA). Gaussian distribution of the data was tested using the d’Agostino–Pearson omnibus normality test. Data are reported as arithmetic mean ± standard deviation. Comparisons between multiple groups were performed by Kruskal–Wallis test, with Dunn’s posttest for non-parametric data. Differences were considered significant at *p* < 0.05.

## 5. Conclusions

The present study did not detect any notable differences between HUVEC and HUAEC regarding selected EC-specific properties necessary for the successful endothelialization of cardiovascular implants, including proliferation, and secretion of vasoactive substances and extracellular matrix proteins. Consequently, EC from both sources can be equally used for in vitro endothelial cell research, despite the fact that cardiovascular implants are mainly applied in the arterial system. This means that study results on implant endothelialization obtained with HUVEC most likely reflect the behavior of arterial EC. In light of potentially applying these findings in preclinical studies, and (while using clinical-grade cell sources) in the future also in clinical applications, it remains to be investigated whether arterial EC from areas that are targets for cardiovascular implants, such as coronary arteries, are superior to umbilical cord-derived blood vessels with regard to the in vitro evaluation of cardiovascular implant materials.

## Data Availability

The data that support the findings of this study are available from the corresponding author upon reasonable request.
